# The influence of age categories on performance parameters during on-court testing in wheelchair basketball players

**DOI:** 10.3389/fspor.2025.1576949

**Published:** 2025-07-18

**Authors:** Lorian Honnorat, Thierry Weissland, Didier Pradon, Ilona Alberca, Jean Romain Rivière, Florian Brassart, Opale Vigié, Arnaud Faupin

**Affiliations:** ^1^ Laboratory Jeunesse—Activité Physique et Sportive—Santé (J-AP2S), University of Toulon, Toulon, France; ^2^ Laboratory Integration from Material to System (IMS), University of Bordeaux, Bordeaux, France; ^3^ Parasport Center—ISPC Synergies, CHU Raymond Poincaré, APHP, Garches, France; ^4^ Pôle P3R—UFAM, Hôpitaux Paris-Est Val-de-Marne, Saint-Maurice, Val-de-Marne, France

**Keywords:** wheelchair basketball, age, sprint, indoor-test, inertial sensor, hierarchical clustering

## Abstract

**Introduction:**

Wheelchair basketball is a highly dynamic sport that requires optimized propulsion techniques, sprint performance, and fatigue resistance. Understanding the biomechanical differences between age groups is crucial for potential estimation and training optimization. This study aimed to analyze the impact of age on sprint performances by comparing junior and senior wheelchair basketball players, while also identifying factors influencing sprint performances beyond age, such as experience, wheelchair characteristics, and classification.

**Materials and methods:**

Twenty-two male wheelchair basketball players were divided into two groups: juniors (21 years or younger) and seniors (22 years and older). Participants completed 6 × 20 m repeated sprint tests, during which various biomechanical parameters including propulsion time, cadence, and asymmetry, were measured using inertial measurement units (IMUs). Fatigue indices were calculated by comparing performance across repeated sprints. Principal component analysis and hierarchical clustering were applied to identify key performance differentiators among groups.

**Results:**

Junior players exhibited significantly lower linear wheelchair velocities from the first three pushes, as well as throughout their best sprint compared to seniors, resulting in significantly longer sprint times in junior players. Additionally, the fatigue index was higher for the junior group. However, no significant differences were found in the stabilized velocity phase, maximum velocity, or propulsion asymmetry of the best sprint. Hierarchical clustering analysis revealed three clusters, with experience and wheel size emerging as additional performance differentiators beyond age.

**Conclusion:**

The study confirms that age influences, on average, sprint performances in wheelchair basketball, particularly in the initial acceleration phase, but the wide interindividual variabilities within age groups were also linked to experience and wheelchair characteristics.

## 1 Introduction

Wheelchair basketball is a highly demanding para-sport that requires amongst other advanced technical skills, and anaerobic capabilities. Due to its high-intensity, intermittent nature, the sport has attracted increasing attention from researchers aiming to optimize performance through evidence-based approaches. Field-based assessments of technical skills and physical capabilities, via single and repeated sprint tests, which have been widely used, such as the 20-meter sprint test to evaluate anaerobic capacity in wheelchair basketball (1–3). These evaluations have shown correlations with athletes' functional classification, wheelchair experience, and training history (4–6), good sensitivity to grasp individual characteristics.

Among the key factors influencing sprint performances, age is particularly significant, notably in young athletes. In para-athletes, short maximal effort performances, such as single sprint time over 100 m, increase with age from early stages of development to age of peak performance (7). Despite being insightful, age-related sprint performances on shorter distances, closer to wheelchair basketball efforts, have never been investigated. It would be of great interest to also study each phase of acceleration from null or low velocities to maximal sprinting velocity, as well as kinematic factors that could explain the associate performance of each phase. Similarly, no previous work has focused on the effect of age on the ability to sprint repeatedly.

Bednarczyk and Sanderson (8) suggest that there are differences in the biomechanics of wheelchair propulsion between children and adults. Propulsion asymmetry is one of the key factors influencing sprint performances, because uneven force application makes steering more complex (9).

One other important factor may be that younger athletes are still in the process of refining their seated posture and adjusting their sport wheelchair configurations, whereas senior players have typically stabilized their setup over years of experience. Such ongoing adaptation in juniors may contribute to greater variability in propulsion patterns and efficiency, and indirectly affect sprint performances. Additionally, Brassart et al. (10) found that these asymmetries are more pronounced in athletes with higher levels of impairment, potentially affecting sprint performances. Taken together, level of impairment and wheelchair characteristics interact with age, affecting sprint performances. Given the multifactorial nature of wheelchair basketball performance, an effective statistical approach would be the Principal Component Analysis (PCA) associated to clustering methods. Indeed, recent works illustrated how the two approaches can reveal nuanced performance profiles beyond traditional functional classes, guiding evidence-based classification and individualized training or equipment prescriptions in para-athletes (11–13).

Understanding age-related variations could help at identifying the specific biomechanical and physiological characteristics of young wheelchair basketball players, as well as potential estimation and training optimization.

The objective of this study was to characterize sprint performances across two separate age categories in wheelchair basketball using field-based tests. A key aspect of this analysis was distinguishing certain kinematic parameters of junior players (aged 21 or younger) that were significantly different from senior players (aged 22 and older).

Our primary hypothesis was that junior players will exhibit lower sprint performances compared to seniors. Additionally, we expect juniors to demonstrate a reduced ability to sustain maximal effort across multiple sprints and greater propulsion asymmetry compared to senior players.

## 2 Materials and methods

### 2.1 Study design

The warm-up consisted of joint mobility exercises followed by five minutes of wheelchair propulsion. The propulsion began at a slow pace, with an increase in frequency every minute. After four minutes, athletes performed two 10-meter sprints to prepare for the test, followed by 30 s of backward rolling for active recovery. The test itself consisted of six sprints from a stationary start, with 20 s of passive recovery between each sprint.

**Figure 1:**
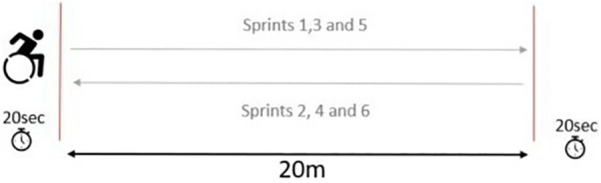
Repeated 6 × 20 m sprint protocol.

After completing the warm-up and receiving a detailed explanation of the test (illustrated in Figure 1), athletes positioned themselves at the starting line. Following a three-second countdown, they propelled forward as quickly as possible and decelerated after crossing the 20-meter mark. They then had 20 s to return to the starting position (which corresponded to the finish line of the previous sprint), maintaining a stationary start for the next sprint. The three-second countdown was repeated before each attempt, and this cycle continued until all six sprints were completed.

Each athlete used their own personalized sports wheelchair, specifically adapted to their needs and the sport. All wheelchairs used in the test had a camber angle between 15° and 22° and wheel sizes ranging from 24 to 27 inches.

To ensure consistency in data collection and allow for accurate comparisons of propulsion parameters, athletes were instructed to use synchronous propulsion throughout the test. All tests were conducted indoors on a parquet floor to maintain uniform surface conditions.

### 2.2 Participants

A total of 22 wheelchair basketball players were included in this study. The inclusion criteria required participants to be male, aged 14 years or older, competing at the national level for at least six months, and having more than two years of experience in wheelchair sports. Athletes reporting any injury or pain that could potentially affect their ability to propel their wheelchair were excluded.

Table 1 presents the characteristics of the participants, divided into two age groups: juniors (21 years or younger) and seniors (22 years and older) (14).

**Table 1:** Participant characteristics.

Groupe	Age (years)	Body mass (kg)	Body height (cm)	BMI (kg/m²)	Classification	Wheel size (inch)	Wheelchair Camber (°)	Years of practice (years)	Handicap
Junior	14	70	165	25.7	3.5	24	18	3	Arthrogryposis scoliosis
Junior	17	113	193	30.3	4.5	26	15	1	Paraplegia
Junior	16	65	175	21.2	4.5	26	18	0.5	Tibial amputation
Junior	14	45	170	15.6	3.5	24	17	1	Cerebral palsy
Junior	16	72	140	36.7	3	26	16	2	Spina bifida
Junior	17	75	165	27.6	2	26	18	3	Paraplegia
Junior	19	50	160	19.5	1.5	26	18	6	Spina bifida
Junior	19	92	170	31.8	4	26	18	4	Incomplete paraplegia
Junior	20	70	140	35.7	4.5	24	18	8	Cerebral palsy
Junior	19	57	180	17.6	3	26	18	7	Cerebral palsy
Junior	19	72	163	27.1	4	27	18	7	Bilateral tibial agenesis
Junior	18	117	172	39.6	4	27	17	7	Leg malformation
Junior	21	67	171	22.9	2.5	26	18	9	Incomplete paraplegia
Junior	21	65	185	19.0	4.5	26	18	8	Neurological epilepsy
Senior	33	80	196	20.8	2.5	27	18	7	Paraplegia
Senior	37	80	184	23.6	3.5	27	18	10	Femoral amputation
Senior	40	80	175	26.1	1	26	18	20	Incomplete paraplegia
Senior	25	63	150	28.0	2.5	25	18	13	Arthrogryposis
Senior	38	85	193	22.8	4.5	26	20	11	Dysplasia
Senior	37	65	180	20.1	1	24	20	13	Paraplegia
Senior	29	63	184	18.6	2.5	27	20	14	Paraplegia
Senior	37	70	171	23.9	3	26	22	23	Poliomyelitis
Mean ± (SD) Junior	17.9 ± (2.3)	73.6 ± (20.8)	167.8 ± (14.7)	26.5 ± (7.6)	3.3 ± (0.8)	25.7 ± (1.0)	17.5 ± (0.9)	5.1 ± (2.8)	
Mean ± (SD) Senior	34.5 ± (5.1)	73.3 ± (9.0)	179.1 ± (14.4)	23.0 ± (3.1)	1.7 ± (1.2)	26.0 ± (1.1)	19.3 ± (1.5)	13.9 ± (5.2)	

SD, standard deviation; BMI, body mass index.

### 2.3 Data measurement

Field data were collected using inertial sensors (IMUs, 128 Hz) equipped with an accelerometer, gyroscope, magnetometer, and Bluetooth module (WheelPerf System, AtoutNovation, France). These sensors were positioned on the axis of each wheel, with the gyroscope used to estimate the wheel's rotational velocity around the z-axis. To ensure accuracy, the camber angle of the wheelchair was considered, in accordance with the method described by Fuss et al. (15). The z-axis was oriented perpendicularly to the plane of the wheels. The IMUs' outputs were validated against a gold-standard optical motion-capture system, demonstrating a mean error of ∼2.5% across various wheelchair-specific key kinematic parameters, including the ones used in the present study (16). Notably, the validation study was conducted using the same IMU devices as those employed in our protocol, thereby supporting the reliability and applicability of the recorded data.

The raw data were filtered using a second-order Butterworth low-pass filter with a cutoff frequency set at 4 Hz. All data processing was performed using MATLAB 2024b.

### 2.4 Outcome parameters

**Figure 2:**
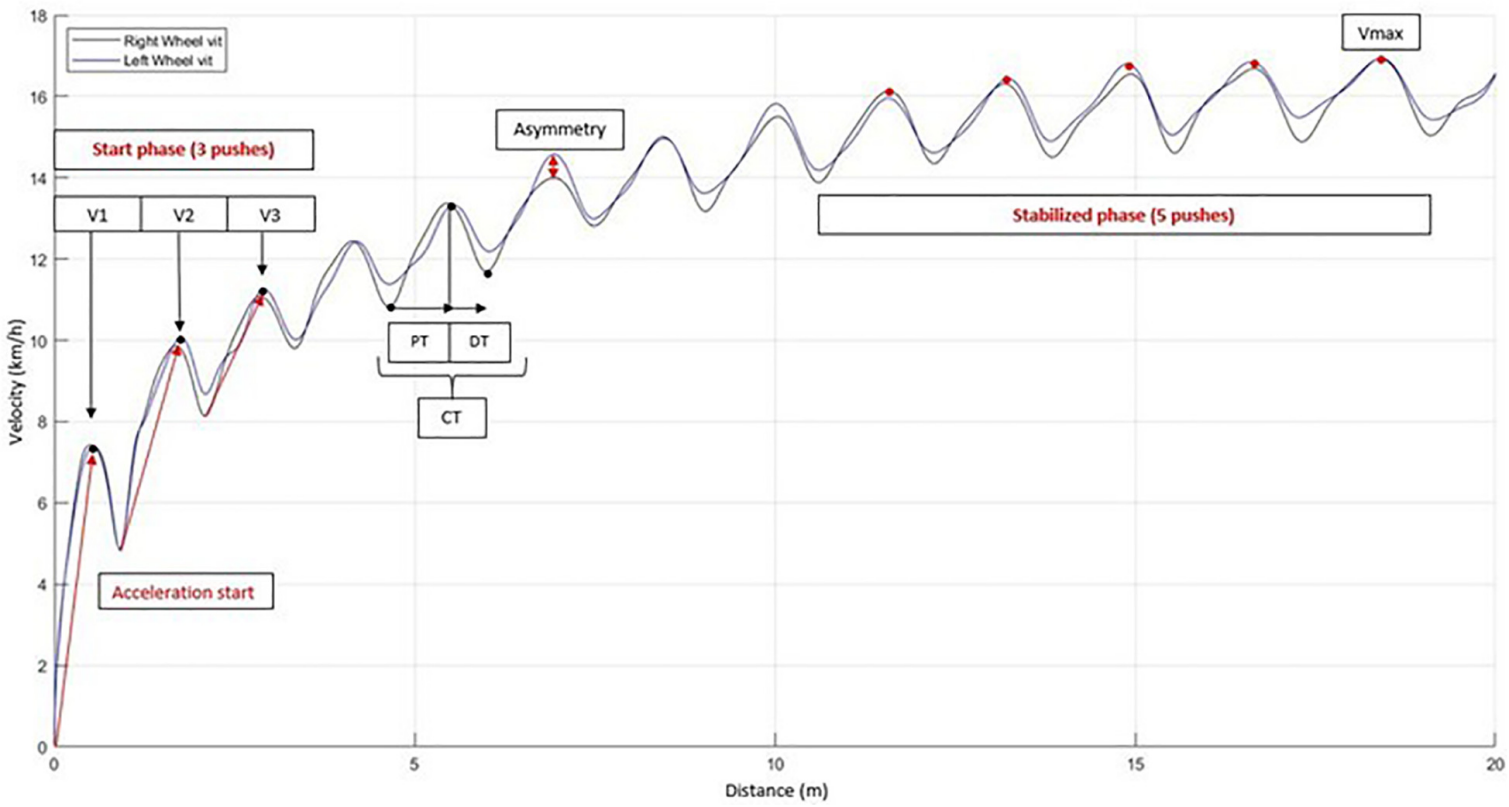
Velocity profiles of both wheels during a 20-meter sprint.

In order to facilitate understanding and interpretation of our experimental measurements, Figure 2 visually summarizes these variables, while Table 2 provides a comprehensive description of each parameter used in the study.

**Table 2:** Description of outcome parameters.

Abbreviation	Parameters	Description
V1	Peak Velocity 1 (m/s)	Peak Velocity for the first push
V2	Peak Velocity 2 (m/s)	Peak Velocity for the second push
V3	Peak Velocity 3 (m/s)	Peak Velocity for the third push
Vend	PeakVelocity Vend (m/s)	Peak Velocity of the last 5 pushes
Vmean	Mean Velocity (m/s)	Mean velocity over the entire sprint
Vmax	Max Velocity (m/s)	Maximum Velocity during the sprint
DVmax	Distance for Vmax (m)	Distance required to reach Vmax
AccStart	Mean Acceleration Start (m/s²)	Mean acceleration for the first 3 pushes
T5 m	Time for 5 m (sec)	Sprint time at 5 meters
T10 m	Time for 10 m (sec)	Sprint time at 10 meters
T15 m	Time for 15 m (sec)	Sprint time at 15 meters
T20 m	Sprint time (sec)	Sprint time at 20 meters
FI_BA	Fatigue Index Best/All (%)	Fatigue Index between the best and the total average of all sprints
PT1	Propulsion phase Time 1 (sec)	Propulsion phase Time of the first push
PT2	Propulsion phase Time 3 (sec)	Propulsion phase Time of the second push
PT3	Propulsion phase Time 3 (sec)	Propulsion phase Time of the third push
PTend	Propulsion phase Time end (sec)	Propulsion phase Time of the last 5 pushes
Asy1	Asymmetry 1 (%)	Difference in velocity between the two wheels for the first push
Asy2	Asymmetry 2 (%)	Difference in velocity between the two wheels for the second push
Asy3	Asymmetry 3 (%)	Difference in velocity between the two wheels for the third push
Asy end	Asymmetry end (%)	Difference in velocity between the two wheels for the last 5 pushes

One fatigue index was selected to ensure a robust assessment of performance decline, independent of sprint order or isolated performance anomalies. This index, previously used by Glaister et al. (17) and Gee et al. (18), was calculated from the sprint times and is defined as follows:

Fatigue Index Best–Average (FI_BA, in %), indicating the deviation from the best sprint to the average sprint performances:

$$FI\_BA=\frac{\left(T_{\mathrm{All}}-T_{\mathrm{Best}}\right)}{T_{\mathrm{Best}}}\times 100$$

Where T_Best_ is the shortest sprint time (best performance) and T_All_ is the mean sprint time across all sprints. This index was chosen over alternative methods (using the last or the slowest sprint time) for several reasons. Firstly, it does not consider the order of the sprints, thus minimizing the impact of transient fluctuations in performance or pacing strategies. Secondly, by relying on the average sprint time rather than the final or worst sprint, the FI provides a more robust and representative measure of the athlete's performance across all sprints. This approach reduces the influence of outlier efforts that may arise from technical errors, temporary discomfort, or other non-fatigue-related issues. Moreover, the normalization to the best sprint time allows for inter-individual comparisons by accounting for each athlete's maximal capacity.

In addition to fatigue index, an asymmetry index was calculated to quantify potential performance imbalances between the left and right wheels (19, 20). Asymmetry Index (in %), representing the relative difference between the faster and slower wheel as follows:

$$\mathrm{Asy}=\frac{\left(\mathrm{Velocit}y_{1}-\mathrm{Velocit}y_{2}\right)}{\mathrm{Velocit}y_{1}}\times 100$$

Where Velocity_1_ is the peak velocity of the wheel with the highest velocity, Velocity_2_ is the peak velocity of the wheel with the lowest velocity. This index reflects the percentage of asymmetry in propulsion, with higher values indicating a greater imbalance between the two wheels.

### 2.5 Statistical methods

#### 2.5.1 Difference of groups

The primary objective was to compare the two age groups based on their sprint performance. The Shapiro–Wilk test was conducted to assess the normality of the data distribution. If a parameter followed a normal distribution, an independent t-test was performed, with a significance threshold set at *p* < 0.05. Cohen's d was used to measure effect size. If a parameter did not follow a normal distribution, a Mann–Whitney independent non-parametric test was applied, with the significance threshold also set at *p* < 0.05. For all non-normally distributed and significant results, the effect size r was calculated using the following equation:

$$r=\frac{Z}{\sqrt{N}}$$

*Where Z represents the statistical result of the test and N is the sample size*.

Effect sizes were classified as small (r = 0.1), moderate (r = 0.3), and large (r = 0.5) (21)

For normally distributed significant results, Cohen's d was calculated using the equation:

$$\mathrm{Cohe}n^{\prime }s\;D=\frac{\left(\mathrm{Mea}n_{\mathrm{Group}\;2}-\mathrm{Mea}n_{\mathrm{Group}1}\right)}{\sqrt{(\sigma_{\mathrm{Group}1}^{2}+\;\sigma_{\mathrm{Group}2}^{2})/2}}$$

*where σ² represents the variance, calculated as the square of the standard deviation*.

The effect sizes were interpreted as small (d < 0.5), moderate (d between 0.50–0.79), and large (d > 0.8) (21).

#### 2.5.2 Hierarchical clustering and PCA analysis

The second objective was to determine whether distinct clusters could be identified based on significant sprint performances. A Principal Component Analysis (PCA) was performed on parameters that showed significant differences between the two age groups.

A hierarchical clustering analysis was conducted based on the coordinates obtained from the principal components. Clustering was performed using an iterative algorithm that merged data points based on Euclidean distances.

Finally, contingency table and boxplot were created to analyze the distribution of age groups within each cluster. The results of the contingency tables are expressed as percentages.

All statistical analyses were performed using R Studio 2024.12.0.

## 3 Results

### 3.1 Impact of age on performance

**Figure 3:**
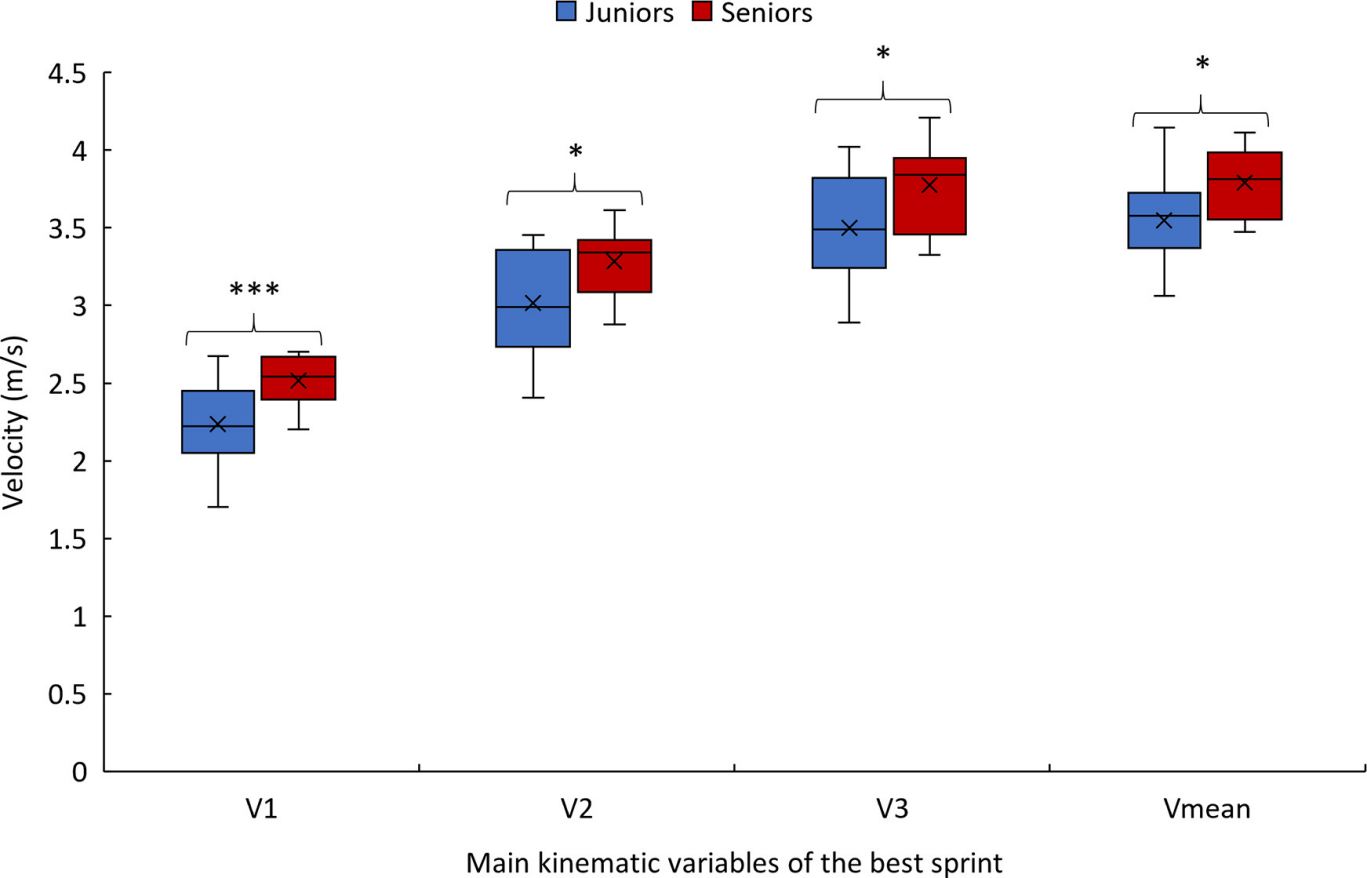
Box plot of velocities used in the PCA for junior and senior. Significance levels: *p* < 0.05 (*); *p* < 0.01 (**); *p* < 0.001 (***).

The comparison of the two age groups is presented in Table 3. Main statistically significant differences in sprint performances between age groups are presented in Figure 3.

**Table 3:** Comparison of athlete characteristics, performance metrics, fatigue index, and propulsion asymmetry between juniors and seniors.

Parameters		Junior Mean (±SD)	Senior Mean (±SD)	*p*	Cohen D or r
Velocity	V1 (m/s)	2.24 (±0.27)	2.52 (±0.18)	**0**.0042***	1.222
	V2 (m/s)	3.02 (±0.34)	3.28 (±0.24)	**0**.0226*	0.904
	V3 (m/s)	3.50 (±0.35)	3.78 (±0.30)	**0**.0355*	0.837
	Vend (m/s)	4.96 (±0.34)	5.17 (±0.33)	0.0922	0.615
	Vmean (m/s)	3.55 (±0.29)	3.79 (±0.23)	**0**.0218*	0.927
	Vmax (m/s)	5.20 (±0.42)	5.43 (±0.38)	0.1014	0.582
Distance	DVmax (m)	18.90 (±0.80)	18.20 (1.21)	0.0872	0.678
Acceleration	AccStart (m/s²)	2.05 (±0.38)	2.31 (±0.35)	0.062	0.71
Times	T5 m (sec)	2.23 (±0.22)	2.04 (±0.15)	**0**.0144*	0.992
	T10 m (sec)	3.49 (±0.32)	3.23 (±0.20)	**0**.0135*	0.998
	T15 m (sec)	4.62 (±0.39)	4.29 (±0.26)	**0**.0144*	0.986
	T20 m (sec)	5.70 (±0.48)	5.31 (±0.32)	**0**.0181*	0.944
Fatigue Index	FI_BA (%)	3.12 (±1.65)	1.88 (±0.95)	**0**.0297*	0.407 (r)
Propulsion Time	PT1 (sec)	0.73 (±0.12)	0.69 (±0.09)	0.749	−0.289
	PT2 (sec)	0.35 (±0.06)	0.33 (±0.04)	0.6465	−0.073 (r)
	PT3 (sec)	0.26 (±0.04)	0.28 (±0.05)	0.2462	0.322
	PTend (sec)	0.20 (±0.03)	0.23 (±0.04)	**0**.0409*	0.867
Asymmetry	Asy1 (%)	4.3 (±4.3)	3.6 (±3.0)	0.5662	−0.029 (r)
	Asy2 (%)	4.6 (±6.0)	3.2 (±3.8)	0.3820	0.073 (r)
	Asy3 (%)	4.2 (±4.3)	3.6 (±2.1)	0.5662	−0.029 (r)
	Asy end (%)	1.8 (±1.7)	1.7 (±1.2)	0.5133	0.000 (r)

SD, standard deviation; *p*, significance threshold set at 0.05 (*), *p* < 0.01 (**), *p* < 0.005 (***); Cohen's d: effect size for parametric values, r: effect size for non-parametric values; significant values are in bold.

### 3.2 Principal component analysis and hierarchical clustering

**Figure 4:**
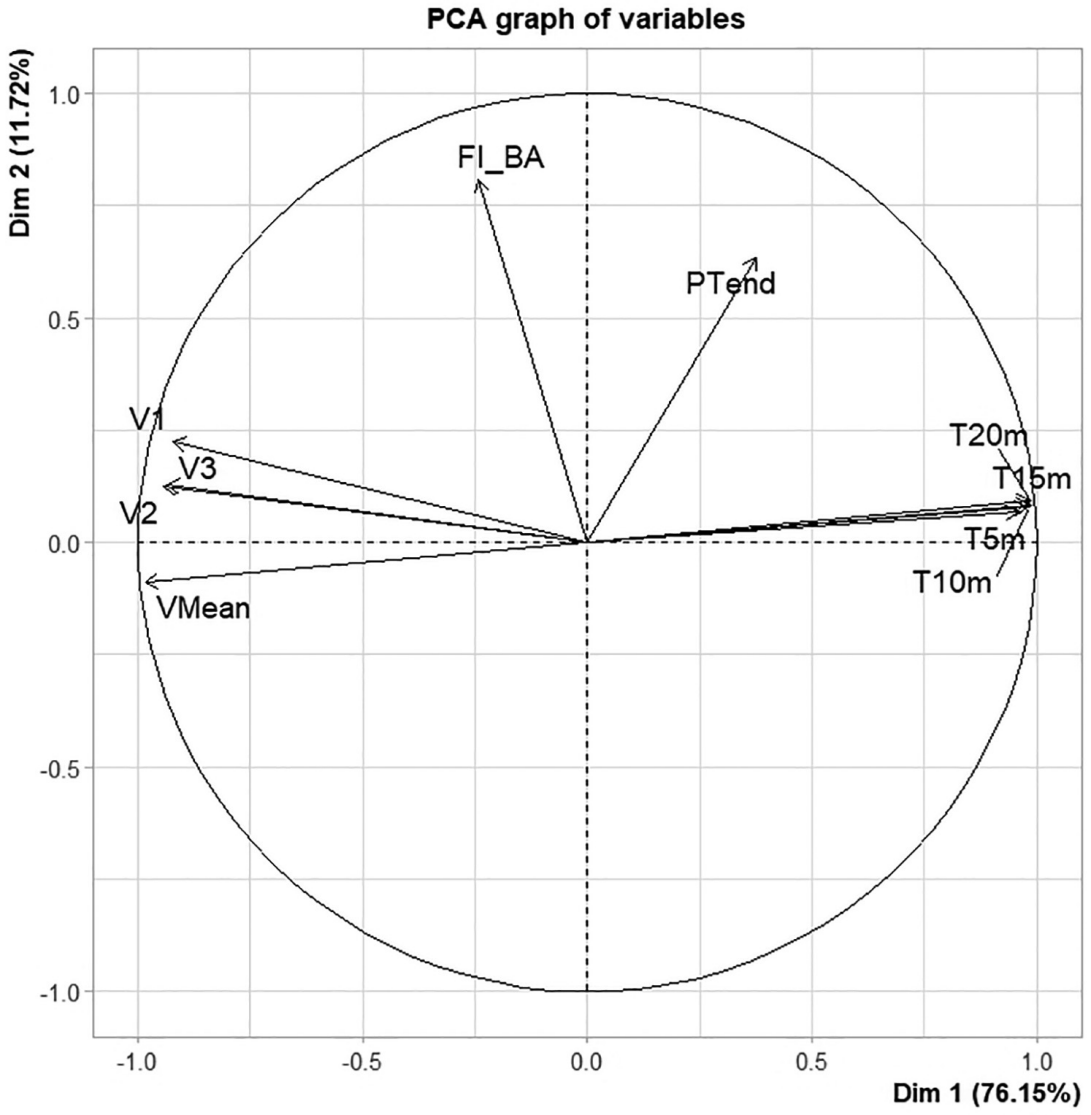
PCA graph of variables on the 2 dimensions. Parameters used in the PCA: V1, V2, V3: velocities of the first three pushes; Vmean: Average sprint velocity; T5 m, T10 m, T15 m and T20 m: Times at 5,10,15 and 20 m; FI_BA, fatigue index between best and all sprints; PTend, propulsion time over the last 5 pushes.

In order to perform hierarchical clustering, PCA is used to obtain the variances of the two principal components and to determine which parameters are associated with each component, presented in Table 4 and in Figure 4.

**Table 4:** PCA summary, including variance explained and variable contributions to each principal component.

Parameters	Dim.1	Dim.2
Variance (%)	76.2	11.7
V1	−0.922	0.225
V2	−0.943	0.125
V3	−0.935	0.129
VMean	−0.984	−0.088
T5 m	0.962	0.068
T10 m	0.982	0.079
T15 m	0.986	0.084
T20 m	0.986	0.092
PTend	0.376	0.635
FI_BA	−0.244	0.807

**Figure 5:**
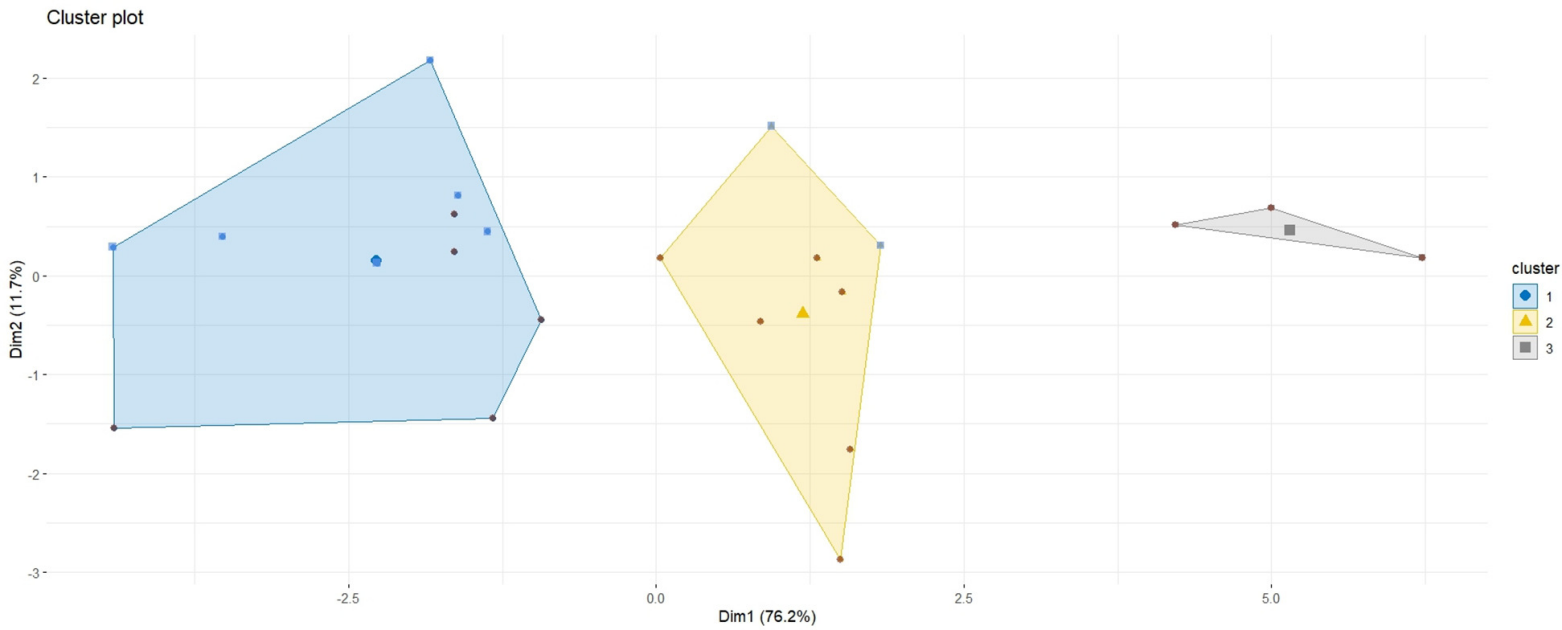
Clustering analysis of each variable along both principal components. Colored dots represent individual athletes: red for juniors and blue for seniors.

**Figure 6:**
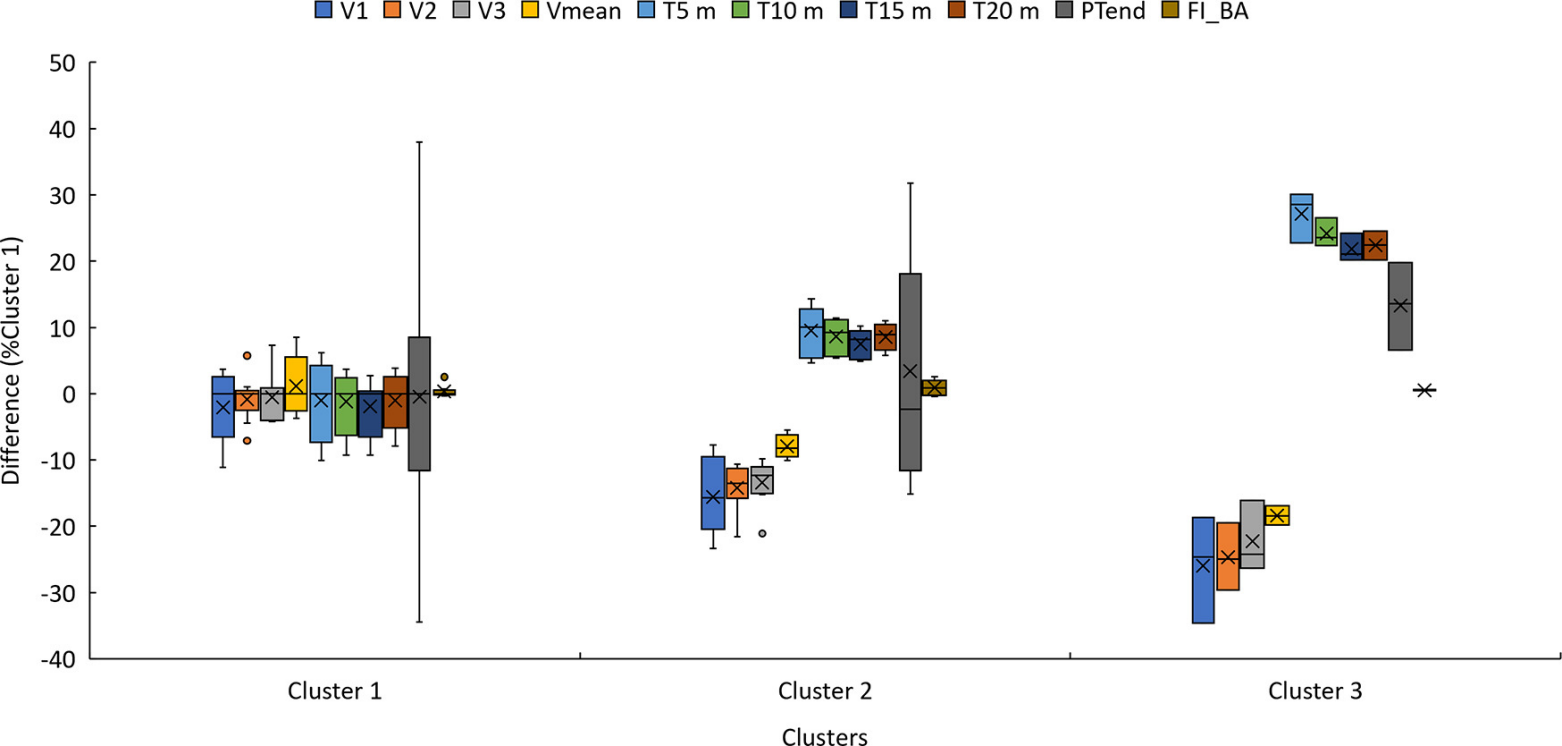
Performance metrics of each cluster expressed relative to the median of cluster 1.

Following the PCA, a hierarchical clustering analysis identified three distinct clusters, as illustrated in Figure 5, with cluster means and standard deviations for all variables presented in Table 5 and Figure 6.

**Table 5:** Clusters means and standard deviations of PCA and supplementary variables.

Parameters	C1	C2	C3
	Mean (±SD)		
V1 (m/s)	2.55 (±0.13)	2.2 (±0.15)	1.93 (±0.21)
V2 (m/s)	3.39 (±0.11)	2.93 (±0.12)	2.58 (±0.17)
V3 (m/s)	3.9 (±0.14)	3.4 (±0.14)	3.05 (±0.21)
Vmean (m/s)	3.86 (±0.16)	3.52 (±0.07)	3.12 (±0.06)
T5 m (sec)	2 (±0.11)	2.22 (±0.08)	2.57 (±0.08)
T10 m (sec)	3.17 (±0.14)	3.49 (±0.09)	3.99 (±0.07)
T15 m (sec)	4.21 (±0.18)	4.62 (±0.09)	5.23 (±0.09)
T20 m (sec)	5.21 (±0.21)	5.71 (±0.1)	6.43 (±0.11)
PTend (sec)	0.2 (±0.04)	0.21 (±0.03)	0.23 (±0.01)
FI_BA (%)	2.28 (±1.39)	3.25 (±1.92)	2.56 (±0.16)
Classification (point)	2.73 (±0.93)	3.69 (±1.22)	3.33 (±1.26)
Experience (year)	10.73 (±6.29)	6.81 (±4.16)	1.67 (±1.15)
Height (cm)	169.64 (±15.96)	173.5 (±16.06)	176 (±14.93)
Weight (kg)	74 (±16.88)	71.12 (±11.61)	77.67 (±34.08)
Wheel size (inch)	26.36 (±0.67)	25.25 (±1.04)	25.33 (±1.15)
Wheel camber (deg)	18.27 (±1.56)	18.5 (±0.93)	16.67 (±1.53)

## 4 Discussion

The results of this study demonstrate significant differences between the two age groups in sprint performance, particularly in sprinting velocity, and fatigue index. These findings highlight the influence of age on sprint performance in wheelchair basketball players, with juniors exhibiting lower overall performance compared to seniors.

The first hypothesis, which posited that sprint times and sprinting velocities would be lower in junior athletes, is partially confirmed. Juniors demonstrated significantly lower sprinting velocities at the beginning of the sprint, average velocity over 20 m, and longer sprint times across all distances (i.e., 5,10,15 and 20 m), which can be observed from the 3 first pushes of the sprint. These results are consistent with previous findings from Bergamini et al. (22), who reported longer sprint durations in younger para-athletes. This performance gap may be partly explained by differences in neuromuscular maturity and biomechanical development. Younger athletes tend to exhibit lower force production, slower rate of force development, and longer electromechanical delays, as shown by Waugh et al. (23), Rumpf et al. (24), and Meyers et al. (25). These factors likely limit the ability of junior players to produce and transfer force effectively during short, explosive efforts such as sprints. Indeed, the discrepancy could stem from lower force output per push, which is consistent with prior findings from Lawton et al. (26) and Gacesa et al. (27), which demonstrated that senior athletes display higher upper limb muscle force production, particularly in the triceps brachii. These findings highlight the importance of developing force-oriented training strategies for younger athletes and optimizing propulsion technique early in their athletic progression to close the performance gap over time. Training for junior athletes should prioritize plyometric drills, sprint-specific techniques, and progressive strength training to improve neuromuscular coordination and force output relative to body weight (25, 28). In comparison, no significant differences were found between age groups in maximum velocity, and stabilized-phase velocity. This suggests that junior athletes are capable of reaching and maintaining maximal sprinting velocity similar to their senior counterparts.

The second hypothesis, which proposed that the fatigue index would be higher for junior players compared to seniors, is validated. This observation aligns with the results reported by Lawton et al. (26) and Gacesa et al. (27), who demonstrated that junior athletes increased upper-body fatigue during performance tests. Interestingly, descriptive data from our sample suggest that junior players tended to have higher classification scores than seniors, reflecting greater functional capacity, which could lead one to expect them to exhibit lower fatigability as reported by Bakatchina et al. (1). One possible explanation for this discrepancy lies in the method used to calculate fatigue. In our study, the index was computed based on the difference between the best sprint and the average of all six sprints, capturing an athlete's ability to sustain high performance across the entire protocol. In contrast, Bakatchina's fatigue index was based on the difference between the best and the last sprint, assuming the final effort represents the lowest performance, an assumption that may not hold true for all athletes, particularly those with irregular pacing or recovery patterns (see Supplementary Figure S1 in Supplementary Material). In particular, these findings raise questions about the suitability of repeated sprint protocols as a unique tool to assess fatigability in youth wheelchair athletes. The larger intra-group variability observed in juniors likely stems from inconsistent technique, pacing, or effort, may inflate fatigue scores independently of actual physiological fatigue. Moreover, repeated sprint tests lack standardized guidelines regarding the number of sprints, recovery durations, and the physiological criteria that define fatigue. Without such consensus, comparing fatigue responses across studies or athlete populations remains problematic. Rather than relying prematurely on a multidimensional fatigue model, these results highlight the need to further investigate how classification interacts with age, training, and technical factors in shaping fatigue responses particularly in youth wheelchair athletes.

The third hypothesis, which proposed that propulsion asymmetry would differ between age groups, was not confirmed. No significant differences in propulsion asymmetry were observed. The lack of significance in our study warrants a more critical examination. One important limitation lies in the nature of the asymmetry index used. This metric considers only the magnitude of peak velocity differences between the left and right wheels, without accounting for temporal discrepancies such as delays between the occurrence of these velocity peaks. As a result, meaningful asymmetries in the timing and coordination of propulsion may go undetected (29). Additionally, individual propulsion styles can vary considerably among athletes especially junior players who are still refining their technique. These variations in motor control and stroke symmetry may have introduced additional variability, potentially masking systematic group-level effects. Another biomechanical factor that may contribute to asymmetry is the phenomenon of steering, defined as involuntary lateral movement of the wheelchair frame during propulsion (9). Steering can occur due to imbalances in push force application, trunk motion, or wheelchair setup, and may generate apparent left–right asymmetries that are not purely muscular or neuromotor in origin. This lateral drift, especially common in dynamic game-like movements, complicates interpretation of kinematic asymmetry indices that do not distinguish between voluntary propulsion and compensatory adjustments. Future studies would benefit from using more comprehensive asymmetry indices that integrate both kinetic and temporal components, allowing for a more nuanced characterization of bilateral propulsion dynamics. Brassart (30) highlighted the influence of trunk movement on wheelchair kinematics, particularly in basketball, where lateral displacement is more pronounced than in rugby. This added dimension of movement may further obscure propulsion asymmetry by diluting clean left–right imbalances. Taken together, these findings suggest that while asymmetry remains a relevant parameter for individualized training and injury prevention, its accurate measurement and interpretation require careful methodological control including consideration of steering effects, propulsion consistency, and equipment setup.

**Figure 7:**
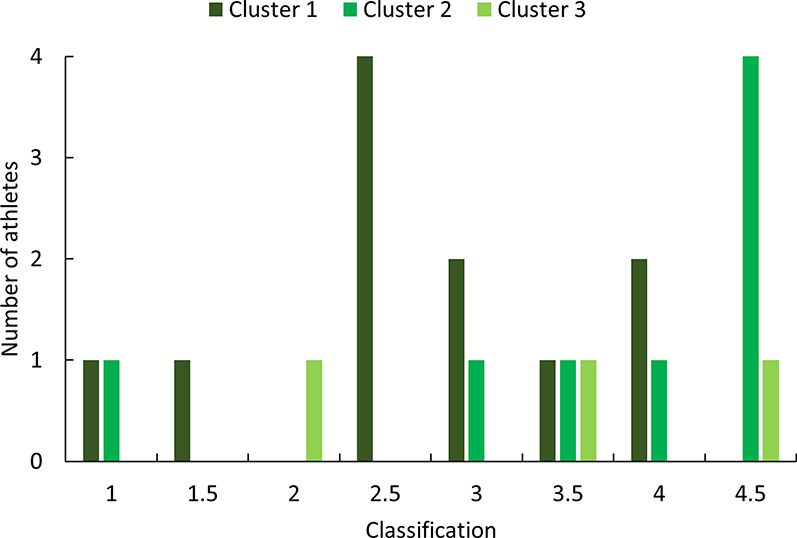
Distribution of classification scores across clusters.

**Figure 8:**
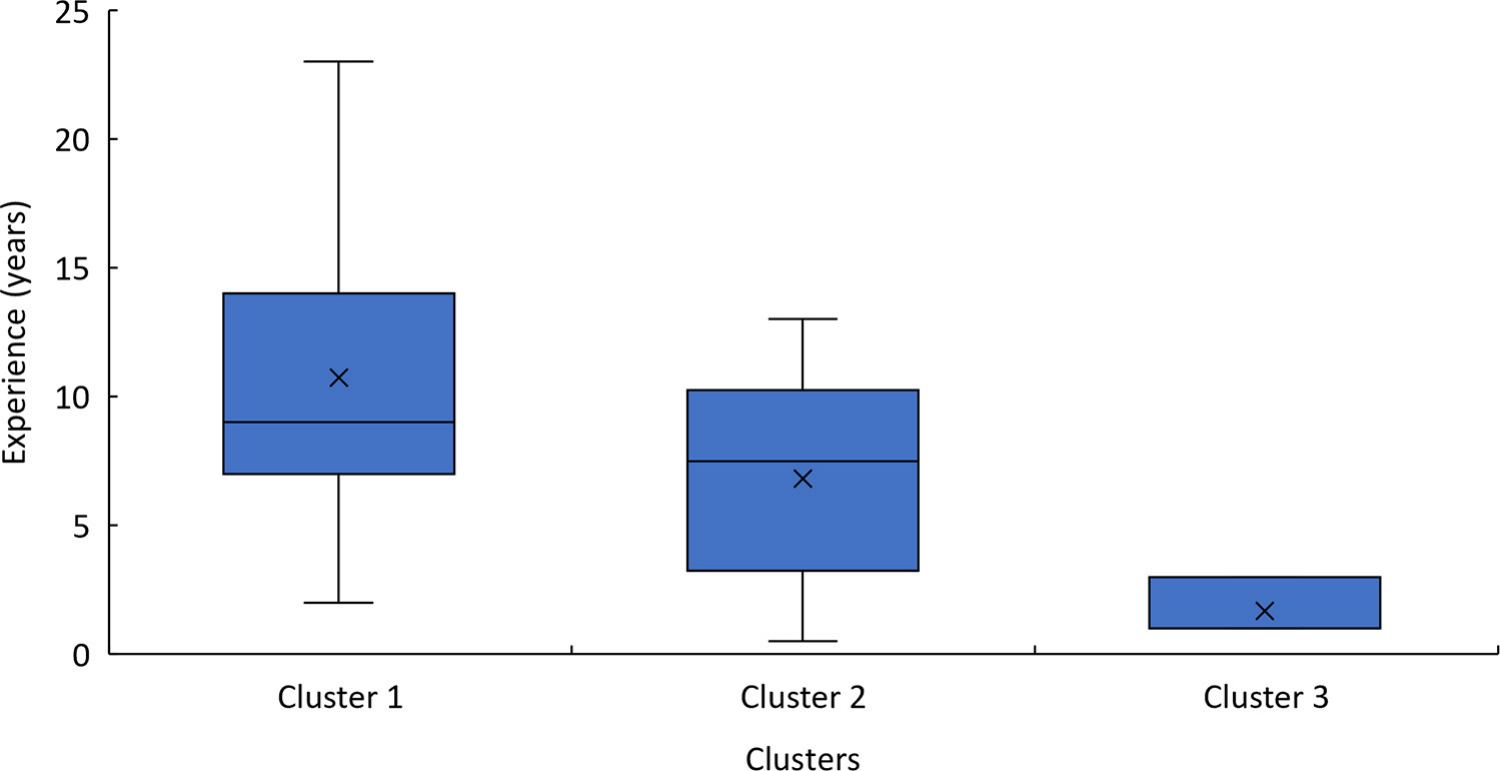
Boxplot of players experience grouped by cluster.

The PCA and hierarchical clustering revealed three distinct groups rather than the expected two, suggesting that factors beyond chronological age could influence performance categorization. The first principal component, which primarily represents sprint times, was the most significant factor distinguishing the clusters. This finding indicates that sprint performance is the primary differentiator among players, regardless of age. This aspect is fundamental in wheelchair basketball for executing quick and efficient actions such as ball possession, defensive transition, and counterattacks. The distribution of players within the clusters further supports the idea that factors beyond age contribute to performance differences. The first cluster contained nearly equal proportions of juniors and seniors, with 45.5% and 54.5%. The second cluster was composed primarily of juniors, with 75% of its members belonging to this group presented in Table 6. Classification scores also varied among clusters, with the second cluster containing a majority of high-point players (87.5%), presented in Figure 7. An athlete classified as “low point” has a classification between 1 and 2.5 points, whereas an athlete classified as “high point” has a classification between 3 and 4.5 points (31). Although classification appears to influence performance, it cannot fully explain the differences observed between and within clusters. Functional classification provides a structured framework to categorize players based on the extent and nature of their physical impairment, primarily focusing on aspects such as muscle strength, joint stability, and motor control in relation to wheelchair-specific functional tasks. While this system is essential for ensuring fairness in competition, it does not directly account for other critical contributors to performance, such as cardiopulmonary capacity, range of motion, or overall mobility. These parameters, although not part of the classification criteria, may act as confounding variables that influence sprint ability, fatigue resistance, and technical execution in field-based settings. Classification thus offers a snapshot of an athlete's functional limitations but may not fully reflect their sport-specific performance potential. Experience, in contrast, reflects the accumulation of technical skills, training exposure, tactical understanding, and equipment familiarity over time. Further analysis of experience levels scores suggests that these factors also play a role in performance differentiation. Almost all players in the first cluster had more than six years of experience, while the third cluster contained only players with less than six years of experience, presented in Figure 8. These results suggest that the positioning of more experienced players in their wheelchairs tends to be more efficient, leading to movement patterns that are better adapted to each athlete. More experienced players are likely to have refined their propulsion technique, adjusted their wheelchair settings, and adapted their training strategies to their own strengths and limitations. Regarding wheelchair characteristics, a more detailed breakdown of wheel size distribution across clusters is presented in Table 7. While players in Cluster 1 predominantly used larger wheels (27 inches in 45.5% of cases), those in Clusters 2 and 3 mostly used 26-inch wheels. Notably, no players in Clusters 2 or 3 used the largest (27-inches) configuration, suggesting a potential link between wheel size and performance grouping.

**Table 6:** Distribution of juniors and seniors within each cluster as percentages.

Clusters	Junior	Senior
Cluster 1	45.5% (5)	54.5% (6)
Cluster 2	75% (6)	25% (2)
Cluster 3	100% (3)	0%

**Table 7:** Distribution of junior and senior within each wheel size group.

Wheel size				
Clusters	24 inches	25 inches	26 inches	27 inches
Cluster 1	0%	9.0% (1)	45.5% (5)	45.5% (5)
Cluster 2	37.5% (3)	0%	62.5% (5)	0%
Cluster 3	33.3% (1)	0%	66.7% (2)	0%

In many cases, they also benefit from custom-fitted equipment, which enhances biomechanical efficiency and comfort. These experiential factors likely contribute to the more effective and consistent performance profiles observed in Cluster 1 players. One visible outcome of this experiential adaptation is the choice of wheelchair configuration particularly wheel size. As previously discussed, larger wheels may offer performance advantages during the stabilized phase of sprinting by improving rolling efficiency (32). However, they also increase mechanical demands during acceleration (33). Interestingly, our results show that players in the most experienced cluster tended to use larger wheels more frequently, suggesting that the benefits of such configurations may be better exploited by players who have developed the requisite strength and technique. Adjusting wheel size may therefore represent a viable performance strategy, but one that should be paired with targeted strength and conditioning work to mitigate the associated force demands.

The potential combined influence of age, experience and wheelchair characteristics may partly explain the emergence of three distinct performance-based clusters rather than a binary age-based separation. These findings emphasize the need to consider equipment configuration, training background and overall experience in athlete assessment and classification frameworks.

From an applied perspective, these clusters can support individualized training strategies and informed equipment selection. For instance, players in the lower-performing cluster (Cluster 2) may benefit from targeted programs focusing on acceleration, and push technique. In contrast, players in Cluster 1 who already use larger wheels may require enhanced upper-body strength and power training to fully exploit their mechanical advantage. Coaches and practitioners can also use cluster profiles to guide wheelchair setup decisions, particularly regarding wheel size, to better align with each athlete's propulsion characteristics and performance goals.

In terms of classification, integrating objective cluster-derived metrics with traditional functional assessments could enhance the fairness and precision of classification processes at the elite level. These findings ultimately strengthen the practical relevance of the study by translating biomechanical insights into actionable recommendations for performance optimization and injury risk reduction.

### 4.1 Limitations and perspectives

This investigation's key strength lies in its commitment to field-based biomechanics: sprint performances were recorded under ecological conditions, ensuring that the resulting clusters reflect authentic athlete behaviors on court. By applying PCA to distill complex kinematic datasets and subsequently performing hierarchical clustering, we revealed three meaningful performance groups rather than the two expected, demonstrating that factors beyond chronological age, such as technical proficiency and tactical experience play pivotal roles in sprint capability. These findings pave the way for more personalized training strategies and equipment choices grounded in real-world data.

However, several limitations must be acknowledged. The sample size was relatively small (22 players), limiting the statistical power and generalizability of the findings, especially in the clustering analysis. Also, years of experience may not reflect the same training regimen for two individuals with the same wheelchair sporting practice, in terms of training intensity and number of sessions, and it also does not take into account years of experience in other wheelchair sports. Moreover, athlete age grouping in this study was based on chronological rather than biological age or neuromuscular maturity, which may vary considerably among younger individuals and influence their physical capacities and performance profiles. Factors such as upper-limb strength, trunk control, and training intensity were not directly measured, and could act as confounding variables influencing fatigue and propulsion technique. The absence of physiological markers, such as heart rate, blood lactate concentration, or ratings of perceived exertion (RPE), further limits the depth of the analysis. These indicators could have helped to distinguish mechanical fatigue (linked to technique or propulsion inefficiency) from metabolic fatigue (associated with physiological overload). Including such physiological data would likely enhance the understanding of repeated sprint ability in this population, providing a clearer picture of individual fatigue responses.

Several directions for future research could enhance the understanding of biomechanical performance in wheelchair basketball. Expanding the study to include female athletes would provide valuable insights into potential gender differences in performance and biomechanics. The inclusion of a more diverse sample would allow for a more comprehensive analysis of factors influencing wheelchair basketball performance.

Additionally, the study sample consisted exclusively of male athletes, which restricts the applicability of findings to female wheelchair basketball players, who may exhibit different biomechanical or physiological characteristics. The absence of sex-based comparison represents a missed opportunity to explore potential gender differences in propulsion and fatigue patterns.

Future studies should aim to address these limitations by incorporating a larger and more diverse sample, including both male and female athletes, and integrating direct physiological measurements alongside biomechanical data. It would also be valuable to assess upper-body strength, muscle activation patterns, and training load history to better understand the interplay between physical capacity and sprint performance. Finally, the use of longitudinal designs could help to capture the developmental trajectory of propulsion efficiency and fatigue resistance in junior athletes as they mature and accumulate experience.

Overall, the findings of this study highlight the importance of considering multiple factors when evaluating wheelchair basketball performance. While age plays a role in determining performance differences, other variables, such as experience, classification, and equipment configuration must also be considered in developing targeted performance strategies and advancing evidence-based classification frameworks. Future research should aim to refine classification methods and develop more targeted training strategies to optimize performance in wheelchair basketball players.

## 5 Conclusion

To conclude, this study highlights that junior players exhibit lower sprint performances than seniors, as evidenced by slower sprinting velocities and longer sprint times from the first pushes. Moreover, the data did not allow for a clear distinction between age groups, since three performance-based clusters emerged instead of two.

These findings suggest that factors beyond age, such as experience, classification, and wheel size can play an important role in performance differences. Given the multitude of elements influencing performance, age alone should not be the sole criterion for athlete evaluation.

Field-based tests employing IMUs enable the analysis of the relationship between players and wheelchair configuration, as well as the interaction and impact of these factors on test performance. The integration of tools such as IMUs into training protocols can facilitate field-based assessments and support the refinement of training programs by coaching staff. Additionally, these tools can offer valuable insights for optimizing wheelchair configurations in real-world settings, particularly when taking the athlete's age into account.

## Data Availability

The raw data supporting the conclusions of this article will be made available by the authors, without undue reservation.
