# Supplementary figures and images for: The influence of age categories on performance parameters during on-court testing in wheelchair basketball players
Source: Front Sports Act Living. 2025 Jul 18;7:1576949. doi: 10.3389/fspor.2025.1576949 (PMC12316469; doi:10.3389/fspor.2025.1576949)

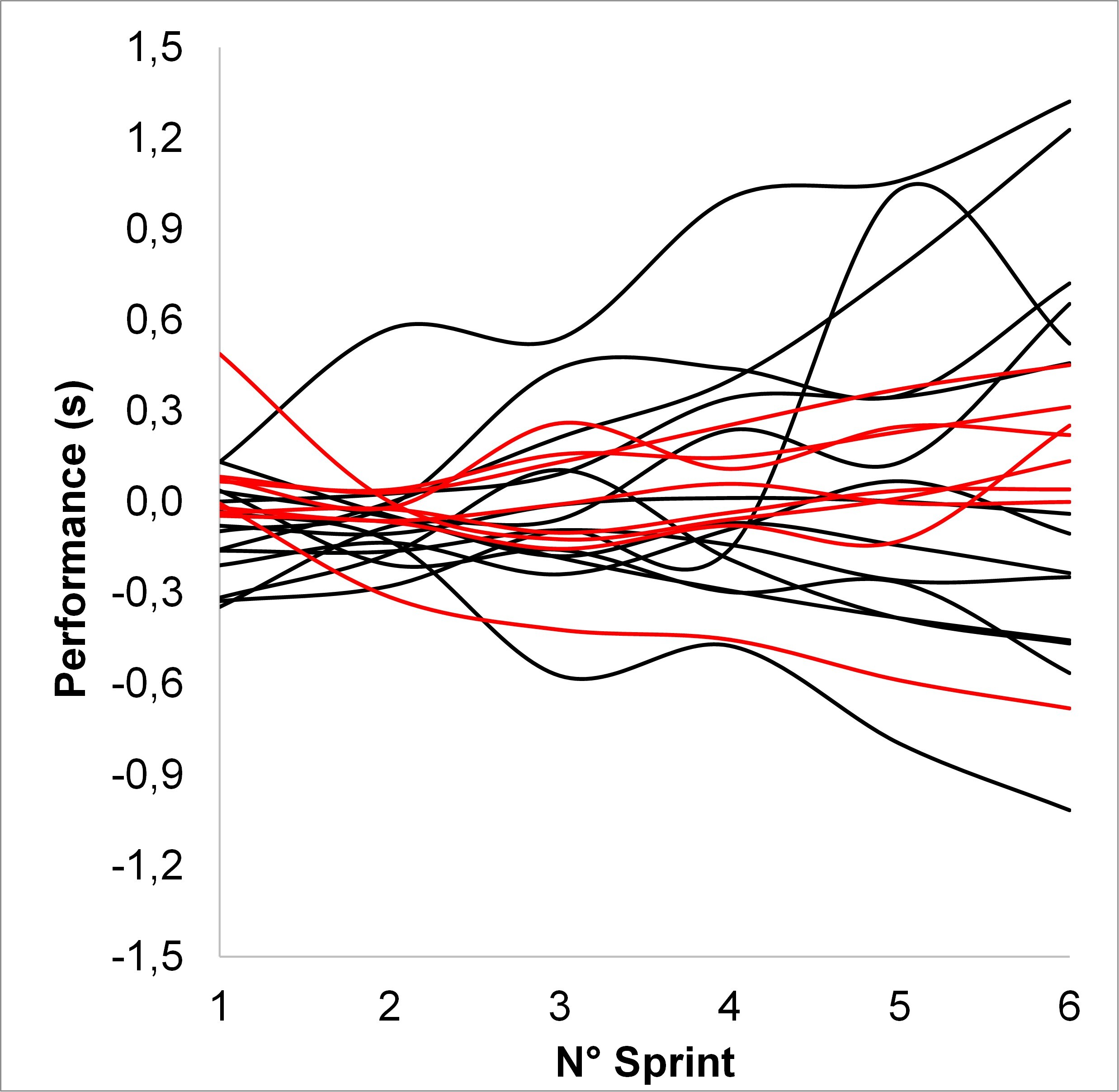

Supplement: Supplementary file 1 [file Image1.jpeg]
